# Supplementary material for: Automatic modular design of robot swarms using behavior trees as a control architecture
Source: PeerJ Comput Sci. 2020 Nov 9;6:e314. doi: 10.7717/peerj-cs.314 (PMC7924474; doi:10.7717/peerj-cs.314)
Supplement: Supplemental Information 3 [file peerj-cs-06-314-s003.zip › NEAT-private-master/misc/config/NetworkGraph/doc.html/Edge.html]

Edge


JavaScript is disabled on your browser.


- Package
- Class
- Tree
- Deprecated
- Index
- Help

- Prev Class
- Next Class

- Frames
- No Frames

- All Classes

- Summary:
- Nested |
- Field |
- Constr |
- Method

- Detail:
- Field |
- Constr |
- Method


## Class Edge

- java.lang.Object
- - Edge

- All Implemented Interfaces:
  :   IEdge

  ---

    

  ```
  public class Edge
  extends java.lang.Object
  implements IEdge
  ```

  Edge Class.

- - ### Constructor Summary

    Constructors

    | Constructor and Description |
    | `Edge(INode n1, INode n2)` |
    | `Edge(INode n1, INode n2, int w)` |
  - ### Method Summary

    Methods

    | Modifier and Type | Method and Description |
    | `boolean` | `contains(INode n)` Checks if the node is at the end of one side of the edge. |
    | `INode` | `getNode1()` Gets the node which is at the end of one side of the edge. |
    | `INode` | `getNode2()` Gets the node which is at the end of the other side of the edge. |
    | `int` | `getWeight()` Gets the weight/cost of the edge. |
    | `void` | `setWeight(int w)` Sets the weight/cost of the edge. |

    - ### Methods inherited from class java.lang.Object

      `clone, equals, finalize, getClass, hashCode, notify, notifyAll, toString, wait, wait, wait`

- - ### Constructor Detail


    - #### Edge

      ```
      public Edge(INode n1,
          INode n2)
      ```


    - #### Edge

      ```
      public Edge(INode n1,
          INode n2,
          int w)
      ```
  - ### Method Detail


    - #### setWeight

      ```
      public void setWeight(int w)
      ```

      **Description copied from interface: `IEdge`**

      Sets the weight/cost of the edge.

      **Specified by:**
      :   `setWeight` in interface `IEdge`

      Parameters:
      :   `w` - weight/cost of the edge.


    - #### getNode1

      ```
      public INode getNode1()
      ```

      **Description copied from interface: `IEdge`**

      Gets the node which is at the end of one side of the edge.

      **Specified by:**
      :   `getNode1` in interface `IEdge`

      Returns:
      :   node1 the first node.


    - #### getNode2

      ```
      public INode getNode2()
      ```

      **Description copied from interface: `IEdge`**

      Gets the node which is at the end of the other side of the edge.

      **Specified by:**
      :   `getNode2` in interface `IEdge`

      Returns:
      :   node2 the 2nd node.


    - #### getWeight

      ```
      public int getWeight()
      ```

      **Description copied from interface: `IEdge`**

      Gets the weight/cost of the edge.

      **Specified by:**
      :   `getWeight` in interface `IEdge`

      Returns:
      :   weight weight/cost of the edge.


    - #### contains

      ```
      public boolean contains(INode n)
      ```

      **Description copied from interface: `IEdge`**

      Checks if the node is at the end of one side of the edge. Comparison between names.

      **Specified by:**
      :   `contains` in interface `IEdge`

      Parameters:
      :   `n` - node to check.

      Returns:
      :   true/false


- Package
- Class
- Tree
- Deprecated
- Index
- Help

- Prev Class
- Next Class

- Frames
- No Frames

- All Classes

- Summary:
- Nested |
- Field |
- Constr |
- Method

- Detail:
- Field |
- Constr |
- Method
