# Supplementary material for: Automatic modular design of robot swarms using behavior trees as a control architecture
Source: PeerJ Comput Sci. 2020 Nov 9;6:e314. doi: 10.7717/peerj-cs.314 (PMC7924474; doi:10.7717/peerj-cs.314)
Supplement: Supplemental Information 3 [file peerj-cs-06-314-s003.zip › NEAT-private-master/misc/config/NetworkGraph/doc.html/Graph.html]

Graph


JavaScript is disabled on your browser.


- Package
- Class
- Tree
- Deprecated
- Index
- Help

- Prev Class
- Next Class

- Frames
- No Frames

- All Classes

- Summary:
- Nested |
- Field |
- Constr |
- Method

- Detail:
- Field |
- Constr |
- Method


## Class Graph

- java.lang.Object
- - Graph

- All Implemented Interfaces:
  :   IGraph

  ---

    

  ```
  public class Graph
  extends java.lang.Object
  implements IGraph
  ```

  Graph Class.

- - ### Constructor Summary

    Constructors

    | Constructor and Description |
    | `Graph()` |
    | `Graph(java.util.ArrayList<INode> ln, java.util.ArrayList<IEdge> le)` |
  - ### Method Summary

    Methods

    | Modifier and Type | Method and Description |
    | `void` | `addEdge(java.util.ArrayList<IEdge> le)` Adds a new list of edges to the graph. |
    | `void` | `addEdge(IEdge e)` Adds a new edge (connecting 2 nodes) to the graph. |
    | `void` | `addNode(java.util.ArrayList<INode> ln)` Adds a new list of nodes to the graph. |
    | `void` | `addNode(INode n)` Adds a new node to the graph. |
    | `int` | `contains(IEdge edge)` Checks if the graph contains a specific edge connecting 2 nodes. |
    | `int` | `contains(INode node)` Checks if the graph contains a specific node. |
    | `IEdge` | `getEdge(int index)` Gets the ith edge. |
    | `java.util.ArrayList<IEdge>` | `getListEdges()` Gets the list of all edges. |
    | `int` | `getListEdgesSize()` Gets the size of the list of all edges. |
    | `java.util.ArrayList<INode>` | `getListNodes()` Gets the list of all nodes. |
    | `int` | `getListNodesSize()` Gets the size of the list of all nodes. |
    | `INode` | `getNode(int index)` Gets the ith node. |
    | `void` | `removeEdge(IEdge e)` Removes an edge from the graph. |
    | `void` | `removeEdge(INode n)` Removes all edges which have a common node at one extremity. |
    | `void` | `removeEdge(int index)` Removes the ith edge from the graph. |
    | `void` | `removeNode(INode n)` Removes a node from the graph. |
    | `void` | `removeNode(int index)` Removes the ith node from the graph. |
    | `void` | `setListNodeAndListEdge(java.util.ArrayList<INode> ln, java.util.ArrayList<IEdge> le)` |
    | `void` | `setPositionToAllNodes(int width, int height)` Sets the position of all nodes in the graph. |

    - ### Methods inherited from class java.lang.Object

      `clone, equals, finalize, getClass, hashCode, notify, notifyAll, toString, wait, wait, wait`

- - ### Constructor Detail


    - #### Graph

      ```
      public Graph(java.util.ArrayList<INode> ln,
           java.util.ArrayList<IEdge> le)
      ```


    - #### Graph

      ```
      public Graph()
      ```
  - ### Method Detail


    - #### setListNodeAndListEdge

      ```
      public void setListNodeAndListEdge(java.util.ArrayList<INode> ln,
                                java.util.ArrayList<IEdge> le)
      ```


    - #### addNode

      ```
      public void addNode(INode n)
      ```

      **Description copied from interface: `IGraph`**

      Adds a new node to the graph.
      If the node has already been defined (i.e. it has the same name with an other node which is
      already in the graph), then it will not be added to the graph.

      **Specified by:**
      :   `addNode` in interface `IGraph`

      Parameters:
      :   `n` - the node.


    - #### addNode

      ```
      public void addNode(java.util.ArrayList<INode> ln)
      ```

      **Description copied from interface: `IGraph`**

      Adds a new list of nodes to the graph.
      If some nodes have already been defined, they won't be added to the graph.

      **Specified by:**
      :   `addNode` in interface `IGraph`

      Parameters:
      :   `ln` - the list of nodes.


    - #### removeNode

      ```
      public void removeNode(INode n)
      ```

      **Description copied from interface: `IGraph`**

      Removes a node from the graph.

      **Specified by:**
      :   `removeNode` in interface `IGraph`

      Parameters:
      :   `n` - the node in question.


    - #### removeNode

      ```
      public void removeNode(int index)
      ```

      **Description copied from interface: `IGraph`**

      Removes the ith node from the graph.

      **Specified by:**
      :   `removeNode` in interface `IGraph`

      Parameters:
      :   `index` - index.


    - #### addEdge

      ```
      public void addEdge(IEdge e)
      ```

      **Description copied from interface: `IGraph`**

      Adds a new edge (connecting 2 nodes) to the graph.
      If the edge has already been defined, it will just change the weight of the edge.

      **Specified by:**
      :   `addEdge` in interface `IGraph`

      Parameters:
      :   `e` - the new edge.


    - #### addEdge

      ```
      public void addEdge(java.util.ArrayList<IEdge> le)
      ```

      **Description copied from interface: `IGraph`**

      Adds a new list of edges to the graph.
      If some edges have already been defined, it will just change the weight of those edges.

      **Specified by:**
      :   `addEdge` in interface `IGraph`

      Parameters:
      :   `le` - the list of edges.


    - #### removeEdge

      ```
      public void removeEdge(IEdge e)
      ```

      **Description copied from interface: `IGraph`**

      Removes an edge from the graph.

      **Specified by:**
      :   `removeEdge` in interface `IGraph`

      Parameters:
      :   `e` - the edge in question.


    - #### removeEdge

      ```
      public void removeEdge(int index)
      ```

      **Description copied from interface: `IGraph`**

      Removes the ith edge from the graph.

      **Specified by:**
      :   `removeEdge` in interface `IGraph`

      Parameters:
      :   `index` - index.


    - #### removeEdge

      ```
      public void removeEdge(INode n)
      ```

      **Description copied from interface: `IGraph`**

      Removes all edges which have a common node at one extremity.

      **Specified by:**
      :   `removeEdge` in interface `IGraph`

      Parameters:
      :   `n` - the common node.


    - #### getNode

      ```
      public INode getNode(int index)
      ```

      **Description copied from interface: `IGraph`**

      Gets the ith node.

      **Specified by:**
      :   `getNode` in interface `IGraph`

      Parameters:
      :   `index` - index.

      Returns:
      :   the ith node.


    - #### getEdge

      ```
      public IEdge getEdge(int index)
      ```

      **Description copied from interface: `IGraph`**

      Gets the ith edge.

      **Specified by:**
      :   `getEdge` in interface `IGraph`

      Parameters:
      :   `index` - index.

      Returns:
      :   the ith edge.


    - #### getListNodes

      ```
      public java.util.ArrayList<INode> getListNodes()
      ```

      **Description copied from interface: `IGraph`**

      Gets the list of all nodes.

      **Specified by:**
      :   `getListNodes` in interface `IGraph`

      Returns:
      :   the list of all nodes.


    - #### getListEdges

      ```
      public java.util.ArrayList<IEdge> getListEdges()
      ```

      **Description copied from interface: `IGraph`**

      Gets the list of all edges.

      **Specified by:**
      :   `getListEdges` in interface `IGraph`

      Returns:
      :   the list of all edges.


    - #### getListNodesSize

      ```
      public int getListNodesSize()
      ```

      **Description copied from interface: `IGraph`**

      Gets the size of the list of all nodes.

      **Specified by:**
      :   `getListNodesSize` in interface `IGraph`

      Returns:
      :   size of the list of all nodes.


    - #### getListEdgesSize

      ```
      public int getListEdgesSize()
      ```

      **Description copied from interface: `IGraph`**

      Gets the size of the list of all edges.

      **Specified by:**
      :   `getListEdgesSize` in interface `IGraph`

      Returns:
      :   size of the list of all edges.


    - #### contains

      ```
      public int contains(INode node)
      ```

      **Description copied from interface: `IGraph`**

      Checks if the graph contains a specific node.

      **Specified by:**
      :   `contains` in interface `IGraph`

      Parameters:
      :   `node` - the specific node in question.

      Returns:
      :   integer. Returns an integer>0 if the graph contains the node.
          In this case, the integer is the index of the node in the graph.
          Returns -1 if the graph doesn't contain the node.


    - #### contains

      ```
      public int contains(IEdge edge)
      ```

      **Description copied from interface: `IGraph`**

      Checks if the graph contains a specific edge connecting 2 nodes.

      **Specified by:**
      :   `contains` in interface `IGraph`

      Parameters:
      :   `edge` - the specific edge in question.

      Returns:
      :   integer. Returns an integer>0 if the graph contains the edge.
          In this case, the integer is the index of the edge in the graph.
          Returns -1 if the graph doesn't contain the edge.


    - #### setPositionToAllNodes

      ```
      public void setPositionToAllNodes(int width,
                               int height)
      ```

      **Description copied from interface: `IGraph`**

      Sets the position of all nodes in the graph.

      **Specified by:**
      :   `setPositionToAllNodes` in interface `IGraph`

      Parameters:
      :   `width` - width of the graph.
      :   `height` - height of the graph.


- Package
- Class
- Tree
- Deprecated
- Index
- Help

- Prev Class
- Next Class

- Frames
- No Frames

- All Classes

- Summary:
- Nested |
- Field |
- Constr |
- Method

- Detail:
- Field |
- Constr |
- Method
