# Supplementary material for: Automatic modular design of robot swarms using behavior trees as a control architecture
Source: PeerJ Comput Sci. 2020 Nov 9;6:e314. doi: 10.7717/peerj-cs.314 (PMC7924474; doi:10.7717/peerj-cs.314)
Supplement: Supplemental Information 3 [file peerj-cs-06-314-s003.zip › NEAT-private-master/misc/config/NetworkGraph/doc.html/GraphPanel.html]

GraphPanel


JavaScript is disabled on your browser.


- Package
- Class
- Tree
- Deprecated
- Index
- Help

- Prev Class
- Next Class

- Frames
- No Frames

- All Classes

- Summary:
- Nested |
- Field |
- Constr |
- Method

- Detail:
- Field |
- Constr |
- Method


## Class GraphPanel

- java.lang.Object
- - java.awt.Component
  - - java.awt.Container
    - - javax.swing.JComponent
      - - javax.swing.JPanel
        - - GraphPanel

- All Implemented Interfaces:
  :   java.awt.event.ActionListener, java.awt.event.MouseListener, java.awt.event.MouseMotionListener, java.awt.image.ImageObserver, java.awt.MenuContainer, java.io.Serializable, java.util.EventListener, javax.accessibility.Accessible

  ---

    

  ```
  public class GraphPanel
  extends javax.swing.JPanel
  implements java.awt.event.MouseListener, java.awt.event.MouseMotionListener, java.awt.event.ActionListener
  ```

  GraphPanel Class.

  See Also:
  :   Serialized Form

- - ### Nested Class Summary

    - ### Nested classes/interfaces inherited from class javax.swing.JPanel

      `javax.swing.JPanel.AccessibleJPanel`
    - ### Nested classes/interfaces inherited from class javax.swing.JComponent

      `javax.swing.JComponent.AccessibleJComponent`
    - ### Nested classes/interfaces inherited from class java.awt.Container

      `java.awt.Container.AccessibleAWTContainer`
    - ### Nested classes/interfaces inherited from class java.awt.Component

      `java.awt.Component.AccessibleAWTComponent, java.awt.Component.BaselineResizeBehavior, java.awt.Component.BltBufferStrategy, java.awt.Component.FlipBufferStrategy`
  - ### Field Summary

    - ### Fields inherited from class javax.swing.JComponent

      `accessibleContext, listenerList, TOOL_TIP_TEXT_KEY, ui, UNDEFINED_CONDITION, WHEN_ANCESTOR_OF_FOCUSED_COMPONENT, WHEN_FOCUSED, WHEN_IN_FOCUSED_WINDOW`
    - ### Fields inherited from class java.awt.Component

      `BOTTOM_ALIGNMENT, CENTER_ALIGNMENT, LEFT_ALIGNMENT, RIGHT_ALIGNMENT, TOP_ALIGNMENT`
    - ### Fields inherited from interface java.awt.image.ImageObserver

      `ABORT, ALLBITS, ERROR, FRAMEBITS, HEIGHT, PROPERTIES, SOMEBITS, WIDTH`
  - ### Constructor Summary

    Constructors

    | Constructor and Description |
    | `GraphPanel(IGraph g)` |
  - ### Method Summary

    Methods

    | Modifier and Type | Method and Description |
    | `void` | `actionPerformed(java.awt.event.ActionEvent e)` |
    | `void` | `addEdge(IEdge edge)` |
    | `void` | `addNode()` |
    | `void` | `closeSocialGraph()` |
    | `void` | `export(java.lang.String format, java.lang.String filePath)` |
    | `void` | `mouseClicked(java.awt.event.MouseEvent e)` |
    | `void` | `mouseDragged(java.awt.event.MouseEvent e)` |
    | `void` | `mouseEntered(java.awt.event.MouseEvent e)` |
    | `void` | `mouseExited(java.awt.event.MouseEvent e)` |
    | `void` | `mouseMoved(java.awt.event.MouseEvent e)` |
    | `void` | `mousePressed(java.awt.event.MouseEvent e)` |
    | `void` | `mouseReleased(java.awt.event.MouseEvent e)` |
    | `void` | `paintComponent(java.awt.Graphics g)` |
    | `void` | `removeEdge(IEdge edge)` |
    | `void` | `removeNode()` |
    | `void` | `zoomIn()` |
    | `void` | `zoomOut()` |

    - ### Methods inherited from class javax.swing.JPanel

      `getAccessibleContext, getUI, getUIClassID, paramString, setUI, updateUI`
    - ### Methods inherited from class javax.swing.JComponent

      `addAncestorListener, addNotify, addVetoableChangeListener, computeVisibleRect, contains, createToolTip, disable, enable, firePropertyChange, firePropertyChange, firePropertyChange, fireVetoableChange, getActionForKeyStroke, getActionMap, getAlignmentX, getAlignmentY, getAncestorListeners, getAutoscrolls, getBaseline, getBaselineResizeBehavior, getBorder, getBounds, getClientProperty, getComponentGraphics, getComponentPopupMenu, getConditionForKeyStroke, getDebugGraphicsOptions, getDefaultLocale, getFontMetrics, getGraphics, getHeight, getInheritsPopupMenu, getInputMap, getInputMap, getInputVerifier, getInsets, getInsets, getListeners, getLocation, getMaximumSize, getMinimumSize, getNextFocusableComponent, getPopupLocation, getPreferredSize, getRegisteredKeyStrokes, getRootPane, getSize, getToolTipLocation, getToolTipText, getToolTipText, getTopLevelAncestor, getTransferHandler, getVerifyInputWhenFocusTarget, getVetoableChangeListeners, getVisibleRect, getWidth, getX, getY, grabFocus, hide, isDoubleBuffered, isLightweightComponent, isManagingFocus, isOpaque, isOptimizedDrawingEnabled, isPaintingForPrint, isPaintingOrigin, isPaintingTile, isRequestFocusEnabled, isValidateRoot, paint, paintBorder, paintChildren, paintImmediately, paintImmediately, print, printAll, printBorder, printChildren, printComponent, processComponentKeyEvent, processKeyBinding, processKeyEvent, processMouseEvent, processMouseMotionEvent, putClientProperty, registerKeyboardAction, registerKeyboardAction, removeAncestorListener, removeNotify, removeVetoableChangeListener, repaint, repaint, requestDefaultFocus, requestFocus, requestFocus, requestFocusInWindow, requestFocusInWindow, resetKeyboardActions, reshape, revalidate, scrollRectToVisible, setActionMap, setAlignmentX, setAlignmentY, setAutoscrolls, setBackground, setBorder, setComponentPopupMenu, setDebugGraphicsOptions, setDefaultLocale, setDoubleBuffered, setEnabled, setFocusTraversalKeys, setFont, setForeground, setInheritsPopupMenu, setInputMap, setInputVerifier, setMaximumSize, setMinimumSize, setNextFocusableComponent, setOpaque, setPreferredSize, setRequestFocusEnabled, setToolTipText, setTransferHandler, setUI, setVerifyInputWhenFocusTarget, setVisible, unregisterKeyboardAction, update`
    - ### Methods inherited from class java.awt.Container

      `add, add, add, add, add, addContainerListener, addImpl, addPropertyChangeListener, addPropertyChangeListener, applyComponentOrientation, areFocusTraversalKeysSet, countComponents, deliverEvent, doLayout, findComponentAt, findComponentAt, getComponent, getComponentAt, getComponentAt, getComponentCount, getComponents, getComponentZOrder, getContainerListeners, getFocusTraversalKeys, getFocusTraversalPolicy, getLayout, getMousePosition, insets, invalidate, isAncestorOf, isFocusCycleRoot, isFocusCycleRoot, isFocusTraversalPolicyProvider, isFocusTraversalPolicySet, layout, list, list, locate, minimumSize, paintComponents, preferredSize, printComponents, processContainerEvent, processEvent, remove, remove, removeAll, removeContainerListener, setComponentZOrder, setFocusCycleRoot, setFocusTraversalPolicy, setFocusTraversalPolicyProvider, setLayout, transferFocusDownCycle, validate, validateTree`
    - ### Methods inherited from class java.awt.Component

      `action, add, addComponentListener, addFocusListener, addHierarchyBoundsListener, addHierarchyListener, addInputMethodListener, addKeyListener, addMouseListener, addMouseMotionListener, addMouseWheelListener, bounds, checkImage, checkImage, coalesceEvents, contains, createImage, createImage, createVolatileImage, createVolatileImage, disableEvents, dispatchEvent, enable, enableEvents, enableInputMethods, firePropertyChange, firePropertyChange, firePropertyChange, firePropertyChange, firePropertyChange, firePropertyChange, getBackground, getBounds, getColorModel, getComponentListeners, getComponentOrientation, getCursor, getDropTarget, getFocusCycleRootAncestor, getFocusListeners, getFocusTraversalKeysEnabled, getFont, getForeground, getGraphicsConfiguration, getHierarchyBoundsListeners, getHierarchyListeners, getIgnoreRepaint, getInputContext, getInputMethodListeners, getInputMethodRequests, getKeyListeners, getLocale, getLocation, getLocationOnScreen, getMouseListeners, getMouseMotionListeners, getMousePosition, getMouseWheelListeners, getName, getParent, getPeer, getPropertyChangeListeners, getPropertyChangeListeners, getSize, getToolkit, getTreeLock, gotFocus, handleEvent, hasFocus, imageUpdate, inside, isBackgroundSet, isCursorSet, isDisplayable, isEnabled, isFocusable, isFocusOwner, isFocusTraversable, isFontSet, isForegroundSet, isLightweight, isMaximumSizeSet, isMinimumSizeSet, isPreferredSizeSet, isShowing, isValid, isVisible, keyDown, keyUp, list, list, list, location, lostFocus, mouseDown, mouseDrag, mouseEnter, mouseExit, mouseMove, mouseUp, move, nextFocus, paintAll, postEvent, prepareImage, prepareImage, processComponentEvent, processFocusEvent, processHierarchyBoundsEvent, processHierarchyEvent, processInputMethodEvent, processMouseWheelEvent, remove, removeComponentListener, removeFocusListener, removeHierarchyBoundsListener, removeHierarchyListener, removeInputMethodListener, removeKeyListener, removeMouseListener, removeMouseMotionListener, removeMouseWheelListener, removePropertyChangeListener, removePropertyChangeListener, repaint, repaint, repaint, resize, resize, setBounds, setBounds, setComponentOrientation, setCursor, setDropTarget, setFocusable, setFocusTraversalKeysEnabled, setIgnoreRepaint, setLocale, setLocation, setLocation, setName, setSize, setSize, show, show, size, toString, transferFocus, transferFocusBackward, transferFocusUpCycle`
    - ### Methods inherited from class java.lang.Object

      `clone, equals, finalize, getClass, hashCode, notify, notifyAll, wait, wait, wait`

- - ### Constructor Detail


    - #### GraphPanel

      ```
      public GraphPanel(IGraph g)
      ```
  - ### Method Detail


    - #### paintComponent

      ```
      public void paintComponent(java.awt.Graphics g)
      ```

      **Overrides:**
      :   `paintComponent` in class `javax.swing.JComponent`


    - #### mouseDragged

      ```
      public void mouseDragged(java.awt.event.MouseEvent e)
      ```

      **Specified by:**
      :   `mouseDragged` in interface `java.awt.event.MouseMotionListener`


    - #### mouseReleased

      ```
      public void mouseReleased(java.awt.event.MouseEvent e)
      ```

      **Specified by:**
      :   `mouseReleased` in interface `java.awt.event.MouseListener`


    - #### mousePressed

      ```
      public void mousePressed(java.awt.event.MouseEvent e)
      ```

      **Specified by:**
      :   `mousePressed` in interface `java.awt.event.MouseListener`


    - #### actionPerformed

      ```
      public void actionPerformed(java.awt.event.ActionEvent e)
      ```

      **Specified by:**
      :   `actionPerformed` in interface `java.awt.event.ActionListener`


    - #### addNode

      ```
      public void addNode()
      ```


    - #### removeNode

      ```
      public void removeNode()
      ```


    - #### addEdge

      ```
      public void addEdge(IEdge edge)
      ```


    - #### removeEdge

      ```
      public void removeEdge(IEdge edge)
      ```


    - #### closeSocialGraph

      ```
      public void closeSocialGraph()
      ```


    - #### export

      ```
      public void export(java.lang.String format,
                java.lang.String filePath)
      ```


    - #### zoomIn

      ```
      public void zoomIn()
      ```


    - #### zoomOut

      ```
      public void zoomOut()
      ```


    - #### mouseMoved

      ```
      public void mouseMoved(java.awt.event.MouseEvent e)
      ```

      **Specified by:**
      :   `mouseMoved` in interface `java.awt.event.MouseMotionListener`


    - #### mouseClicked

      ```
      public void mouseClicked(java.awt.event.MouseEvent e)
      ```

      **Specified by:**
      :   `mouseClicked` in interface `java.awt.event.MouseListener`


    - #### mouseEntered

      ```
      public void mouseEntered(java.awt.event.MouseEvent e)
      ```

      **Specified by:**
      :   `mouseEntered` in interface `java.awt.event.MouseListener`


    - #### mouseExited

      ```
      public void mouseExited(java.awt.event.MouseEvent e)
      ```

      **Specified by:**
      :   `mouseExited` in interface `java.awt.event.MouseListener`


- Package
- Class
- Tree
- Deprecated
- Index
- Help

- Prev Class
- Next Class

- Frames
- No Frames

- All Classes

- Summary:
- Nested |
- Field |
- Constr |
- Method

- Detail:
- Field |
- Constr |
- Method
