# Supplementary material for: Automatic modular design of robot swarms using behavior trees as a control architecture
Source: PeerJ Comput Sci. 2020 Nov 9;6:e314. doi: 10.7717/peerj-cs.314 (PMC7924474; doi:10.7717/peerj-cs.314)
Supplement: Supplemental Information 3 [file peerj-cs-06-314-s003.zip › NEAT-private-master/misc/config/NetworkGraph/doc.html/IGraph.html]

IGraph


JavaScript is disabled on your browser.


- Package
- Class
- Tree
- Deprecated
- Index
- Help

- Prev Class
- Next Class

- Frames
- No Frames

- All Classes

- Summary:
- Nested |
- Field |
- Constr |
- Method

- Detail:
- Field |
- Constr |
- Method


## Interface IGraph

- All Known Implementing Classes:
  :   Graph

  ---

    

  ```
  public interface IGraph
  ```

  Graph Interface.

- - ### Method Summary

    Methods

    | Modifier and Type | Method and Description |
    | `void` | `addEdge(java.util.ArrayList<IEdge> listEdge)` Adds a new list of edges to the graph. |
    | `void` | `addEdge(IEdge edge)` Adds a new edge (connecting 2 nodes) to the graph. |
    | `void` | `addNode(java.util.ArrayList<INode> listNode)` Adds a new list of nodes to the graph. |
    | `void` | `addNode(INode node)` Adds a new node to the graph. |
    | `int` | `contains(IEdge edge)` Checks if the graph contains a specific edge connecting 2 nodes. |
    | `int` | `contains(INode node)` Checks if the graph contains a specific node. |
    | `IEdge` | `getEdge(int i)` Gets the ith edge. |
    | `java.util.ArrayList<IEdge>` | `getListEdges()` Gets the list of all edges. |
    | `int` | `getListEdgesSize()` Gets the size of the list of all edges. |
    | `java.util.ArrayList<INode>` | `getListNodes()` Gets the list of all nodes. |
    | `int` | `getListNodesSize()` Gets the size of the list of all nodes. |
    | `INode` | `getNode(int i)` Gets the ith node. |
    | `void` | `removeEdge(IEdge edge)` Removes an edge from the graph. |
    | `void` | `removeEdge(INode node)` Removes all edges which have a common node at one extremity. |
    | `void` | `removeEdge(int i)` Removes the ith edge from the graph. |
    | `void` | `removeNode(INode node)` Removes a node from the graph. |
    | `void` | `removeNode(int i)` Removes the ith node from the graph. |
    | `void` | `setPositionToAllNodes(int width, int height)` Sets the position of all nodes in the graph. |

- - ### Method Detail


    - #### addNode

      ```
      void addNode(INode node)
      ```

      Adds a new node to the graph.
      If the node has already been defined (i.e. it has the same name with an other node which is
      already in the graph), then it will not be added to the graph.

      Parameters:
      :   `node` - the node.


    - #### addNode

      ```
      void addNode(java.util.ArrayList<INode> listNode)
      ```

      Adds a new list of nodes to the graph.
      If some nodes have already been defined, they won't be added to the graph.

      Parameters:
      :   `listNode` - the list of nodes.


    - #### removeNode

      ```
      void removeNode(INode node)
      ```

      Removes a node from the graph.

      Parameters:
      :   `node` - the node in question.


    - #### removeNode

      ```
      void removeNode(int i)
      ```

      Removes the ith node from the graph.

      Parameters:
      :   `i` - index.


    - #### addEdge

      ```
      void addEdge(IEdge edge)
      ```

      Adds a new edge (connecting 2 nodes) to the graph.
      If the edge has already been defined, it will just change the weight of the edge.

      Parameters:
      :   `edge` - the new edge.


    - #### addEdge

      ```
      void addEdge(java.util.ArrayList<IEdge> listEdge)
      ```

      Adds a new list of edges to the graph.
      If some edges have already been defined, it will just change the weight of those edges.

      Parameters:
      :   `listEdge` - the list of edges.


    - #### removeEdge

      ```
      void removeEdge(IEdge edge)
      ```

      Removes an edge from the graph.

      Parameters:
      :   `edge` - the edge in question.


    - #### removeEdge

      ```
      void removeEdge(int i)
      ```

      Removes the ith edge from the graph.

      Parameters:
      :   `i` - index.


    - #### removeEdge

      ```
      void removeEdge(INode node)
      ```

      Removes all edges which have a common node at one extremity.

      Parameters:
      :   `node` - the common node.


    - #### getNode

      ```
      INode getNode(int i)
      ```

      Gets the ith node.

      Parameters:
      :   `i` - index.

      Returns:
      :   the ith node.


    - #### getEdge

      ```
      IEdge getEdge(int i)
      ```

      Gets the ith edge.

      Parameters:
      :   `i` - index.

      Returns:
      :   the ith edge.


    - #### getListNodes

      ```
      java.util.ArrayList<INode> getListNodes()
      ```

      Gets the list of all nodes.

      Returns:
      :   the list of all nodes.


    - #### getListEdges

      ```
      java.util.ArrayList<IEdge> getListEdges()
      ```

      Gets the list of all edges.

      Returns:
      :   the list of all edges.


    - #### getListNodesSize

      ```
      int getListNodesSize()
      ```

      Gets the size of the list of all nodes.

      Returns:
      :   size of the list of all nodes.


    - #### getListEdgesSize

      ```
      int getListEdgesSize()
      ```

      Gets the size of the list of all edges.

      Returns:
      :   size of the list of all edges.


    - #### contains

      ```
      int contains(INode node)
      ```

      Checks if the graph contains a specific node.

      Parameters:
      :   `node` - the specific node in question.

      Returns:
      :   integer. Returns an integer>0 if the graph contains the node.
          In this case, the integer is the index of the node in the graph.
          Returns -1 if the graph doesn't contain the node.


    - #### contains

      ```
      int contains(IEdge edge)
      ```

      Checks if the graph contains a specific edge connecting 2 nodes.

      Parameters:
      :   `edge` - the specific edge in question.

      Returns:
      :   integer. Returns an integer>0 if the graph contains the edge.
          In this case, the integer is the index of the edge in the graph.
          Returns -1 if the graph doesn't contain the edge.


    - #### setPositionToAllNodes

      ```
      void setPositionToAllNodes(int width,
                               int height)
      ```

      Sets the position of all nodes in the graph.

      Parameters:
      :   `width` - width of the graph.
      :   `height` - height of the graph.


- Package
- Class
- Tree
- Deprecated
- Index
- Help

- Prev Class
- Next Class

- Frames
- No Frames

- All Classes

- Summary:
- Nested |
- Field |
- Constr |
- Method

- Detail:
- Field |
- Constr |
- Method
