# Supplementary material for: Automatic modular design of robot swarms using behavior trees as a control architecture
Source: PeerJ Comput Sci. 2020 Nov 9;6:e314. doi: 10.7717/peerj-cs.314 (PMC7924474; doi:10.7717/peerj-cs.314)
Supplement: Supplemental Information 3 [file peerj-cs-06-314-s003.zip › NEAT-private-master/misc/config/NetworkGraph/doc.html/INode.Type.html]

INode.Type


JavaScript is disabled on your browser.


- Package
- Class
- Tree
- Deprecated
- Index
- Help

- Prev Class
- Next Class

- Frames
- No Frames

- All Classes

- Summary:
- Nested |
- Enum Constants |
- Field |
- Method

- Detail:
- Enum Constants |
- Field |
- Method


## Enum INode.Type

- java.lang.Object
- - java.lang.Enum<INode.Type>
  - - INode.Type

- All Implemented Interfaces:
  :   java.io.Serializable, java.lang.Comparable<INode.Type>

  Enclosing interface:
  :   INode

  ---

    

  ```
  public static enum INode.Type
  extends java.lang.Enum<INode.Type>
  ```

- - ### Enum Constant Summary

    Enum Constants

    | Enum Constant and Description |
    | `HIDDEN` |
    | `INPUT` |
    | `OUTPUT` |
  - ### Method Summary

    Methods

    | Modifier and Type | Method and Description |
    | `static INode.Type` | `valueOf(java.lang.String name)` Returns the enum constant of this type with the specified name. |
    | `static INode.Type[]` | `values()` Returns an array containing the constants of this enum type, in the order they are declared. |

    - ### Methods inherited from class java.lang.Enum

      `clone, compareTo, equals, finalize, getDeclaringClass, hashCode, name, ordinal, toString, valueOf`
    - ### Methods inherited from class java.lang.Object

      `getClass, notify, notifyAll, wait, wait, wait`

- - ### Enum Constant Detail


    - #### INPUT

      ```
      public static final INode.Type INPUT
      ```


    - #### HIDDEN

      ```
      public static final INode.Type HIDDEN
      ```


    - #### OUTPUT

      ```
      public static final INode.Type OUTPUT
      ```
  - ### Method Detail


    - #### values

      ```
      public static INode.Type[] values()
      ```

      Returns an array containing the constants of this enum type, in
      the order they are declared. This method may be used to iterate
      over the constants as follows:

      ```
      for (INode.Type c : INode.Type.values())
          System.out.println(c);
      ```

      Returns:
      :   an array containing the constants of this enum type, in the order they are declared


    - #### valueOf

      ```
      public static INode.Type valueOf(java.lang.String name)
      ```

      Returns the enum constant of this type with the specified name.
      The string must match *exactly* an identifier used to declare an
      enum constant in this type. (Extraneous whitespace characters are
      not permitted.)

      Parameters:
      :   `name` - the name of the enum constant to be returned.

      Returns:
      :   the enum constant with the specified name

      Throws:
      :   `java.lang.IllegalArgumentException` - if this enum type has no constant with the specified name
      :   `java.lang.NullPointerException` - if the argument is null


- Package
- Class
- Tree
- Deprecated
- Index
- Help

- Prev Class
- Next Class

- Frames
- No Frames

- All Classes

- Summary:
- Nested |
- Enum Constants |
- Field |
- Method

- Detail:
- Enum Constants |
- Field |
- Method
