# Supplementary material for: Automatic modular design of robot swarms using behavior trees as a control architecture
Source: PeerJ Comput Sci. 2020 Nov 9;6:e314. doi: 10.7717/peerj-cs.314 (PMC7924474; doi:10.7717/peerj-cs.314)
Supplement: Supplemental Information 3 [file peerj-cs-06-314-s003.zip › NEAT-private-master/misc/config/NetworkGraph/doc.html/Main.html]

Main


JavaScript is disabled on your browser.


- Package
- Class
- Tree
- Deprecated
- Index
- Help

- Prev Class
- Next Class

- Frames
- No Frames

- All Classes

- Summary:
- Nested |
- Field |
- Constr |
- Method

- Detail:
- Field |
- Constr |
- Method


## Class Main

- java.lang.Object
- - Main

- ---

    

  ```
  public class Main
  extends java.lang.Object
  ```

  Main Class.

- - ### Constructor Summary

    Constructors

    | Constructor and Description |
    | `Main()` |
  - ### Method Summary

    Methods

    | Modifier and Type | Method and Description |
    | `static void` | `main(java.lang.String[] args)` |

    - ### Methods inherited from class java.lang.Object

      `clone, equals, finalize, getClass, hashCode, notify, notifyAll, toString, wait, wait, wait`

- - ### Constructor Detail


    - #### Main

      ```
      public Main()
      ```
  - ### Method Detail


    - #### main

      ```
      public static void main(java.lang.String[] args)
      ```

      Parameters:
      :   `args` -


- Package
- Class
- Tree
- Deprecated
- Index
- Help

- Prev Class
- Next Class

- Frames
- No Frames

- All Classes

- Summary:
- Nested |
- Field |
- Constr |
- Method

- Detail:
- Field |
- Constr |
- Method
