# Supplementary material for: Automatic modular design of robot swarms using behavior trees as a control architecture
Source: PeerJ Comput Sci. 2020 Nov 9;6:e314. doi: 10.7717/peerj-cs.314 (PMC7924474; doi:10.7717/peerj-cs.314)
Supplement: Supplemental Information 3 [file peerj-cs-06-314-s003.zip › NEAT-private-master/misc/config/NetworkGraph/doc.html/NNFrame.html]

NNFrame


JavaScript is disabled on your browser.


- Package
- Class
- Tree
- Deprecated
- Index
- Help

- Prev Class
- Next Class

- Frames
- No Frames

- All Classes

- Summary:
- Nested |
- Field |
- Constr |
- Method

- Detail:
- Field |
- Constr |
- Method


## Class NNFrame

- java.lang.Object
- - java.awt.Component
  - - java.awt.Container
    - - java.awt.Window
      - - java.awt.Frame
        - - javax.swing.JFrame
          - - NNFrame

- All Implemented Interfaces:
  :   java.awt.event.ActionListener, java.awt.event.MouseListener, java.awt.image.ImageObserver, java.awt.MenuContainer, java.io.Serializable, java.util.EventListener, javax.accessibility.Accessible, javax.swing.event.MenuListener, javax.swing.RootPaneContainer, javax.swing.WindowConstants

  ---

    

  ```
  public class NNFrame
  extends javax.swing.JFrame
  implements java.awt.event.ActionListener, java.awt.event.MouseListener, javax.swing.event.MenuListener
  ```

  NNFrame (=Neural Network Frame) Class.

  See Also:
  :   Serialized Form

- - ### Nested Class Summary

    - ### Nested classes/interfaces inherited from class javax.swing.JFrame

      `javax.swing.JFrame.AccessibleJFrame`
    - ### Nested classes/interfaces inherited from class java.awt.Frame

      `java.awt.Frame.AccessibleAWTFrame`
    - ### Nested classes/interfaces inherited from class java.awt.Window

      `java.awt.Window.AccessibleAWTWindow, java.awt.Window.Type`
    - ### Nested classes/interfaces inherited from class java.awt.Container

      `java.awt.Container.AccessibleAWTContainer`
    - ### Nested classes/interfaces inherited from class java.awt.Component

      `java.awt.Component.AccessibleAWTComponent, java.awt.Component.BaselineResizeBehavior, java.awt.Component.BltBufferStrategy, java.awt.Component.FlipBufferStrategy`
  - ### Field Summary

    - ### Fields inherited from class javax.swing.JFrame

      `accessibleContext, EXIT_ON_CLOSE, rootPane, rootPaneCheckingEnabled`
    - ### Fields inherited from class java.awt.Frame

      `CROSSHAIR_CURSOR, DEFAULT_CURSOR, E_RESIZE_CURSOR, HAND_CURSOR, ICONIFIED, MAXIMIZED_BOTH, MAXIMIZED_HORIZ, MAXIMIZED_VERT, MOVE_CURSOR, N_RESIZE_CURSOR, NE_RESIZE_CURSOR, NORMAL, NW_RESIZE_CURSOR, S_RESIZE_CURSOR, SE_RESIZE_CURSOR, SW_RESIZE_CURSOR, TEXT_CURSOR, W_RESIZE_CURSOR, WAIT_CURSOR`
    - ### Fields inherited from class java.awt.Component

      `BOTTOM_ALIGNMENT, CENTER_ALIGNMENT, LEFT_ALIGNMENT, RIGHT_ALIGNMENT, TOP_ALIGNMENT`
    - ### Fields inherited from interface javax.swing.WindowConstants

      `DISPOSE_ON_CLOSE, DO_NOTHING_ON_CLOSE, HIDE_ON_CLOSE`
    - ### Fields inherited from interface java.awt.image.ImageObserver

      `ABORT, ALLBITS, ERROR, FRAMEBITS, HEIGHT, PROPERTIES, SOMEBITS, WIDTH`
  - ### Constructor Summary

    Constructors

    | Constructor and Description |
    | `NNFrame(GraphPanel p)` |
  - ### Method Summary

    Methods

    | Modifier and Type | Method and Description |
    | `void` | `actionPerformed(java.awt.event.ActionEvent e)` |
    | `void` | `menuCanceled(javax.swing.event.MenuEvent e)` |
    | `void` | `menuDeselected(javax.swing.event.MenuEvent e)` |
    | `void` | `menuSelected(javax.swing.event.MenuEvent e)` |
    | `void` | `mouseClicked(java.awt.event.MouseEvent arg0)` |
    | `void` | `mouseEntered(java.awt.event.MouseEvent arg0)` |
    | `void` | `mouseExited(java.awt.event.MouseEvent arg0)` |
    | `void` | `mousePressed(java.awt.event.MouseEvent arg0)` |
    | `void` | `mouseReleased(java.awt.event.MouseEvent arg0)` |

    - ### Methods inherited from class javax.swing.JFrame

      `addImpl, createRootPane, frameInit, getAccessibleContext, getContentPane, getDefaultCloseOperation, getGlassPane, getGraphics, getJMenuBar, getLayeredPane, getRootPane, getTransferHandler, isDefaultLookAndFeelDecorated, isRootPaneCheckingEnabled, paramString, processWindowEvent, remove, repaint, setContentPane, setDefaultCloseOperation, setDefaultLookAndFeelDecorated, setGlassPane, setIconImage, setJMenuBar, setLayeredPane, setLayout, setRootPane, setRootPaneCheckingEnabled, setTransferHandler, update`
    - ### Methods inherited from class java.awt.Frame

      `addNotify, getCursorType, getExtendedState, getFrames, getIconImage, getMaximizedBounds, getMenuBar, getState, getTitle, isResizable, isUndecorated, remove, removeNotify, setBackground, setCursor, setExtendedState, setMaximizedBounds, setMenuBar, setOpacity, setResizable, setShape, setState, setTitle, setUndecorated`
    - ### Methods inherited from class java.awt.Window

      `addPropertyChangeListener, addPropertyChangeListener, addWindowFocusListener, addWindowListener, addWindowStateListener, applyResourceBundle, applyResourceBundle, createBufferStrategy, createBufferStrategy, dispose, getBackground, getBufferStrategy, getFocusableWindowState, getFocusCycleRootAncestor, getFocusOwner, getFocusTraversalKeys, getIconImages, getInputContext, getListeners, getLocale, getModalExclusionType, getMostRecentFocusOwner, getOpacity, getOwnedWindows, getOwner, getOwnerlessWindows, getShape, getToolkit, getType, getWarningString, getWindowFocusListeners, getWindowListeners, getWindows, getWindowStateListeners, hide, isActive, isAlwaysOnTop, isAlwaysOnTopSupported, isAutoRequestFocus, isFocusableWindow, isFocusCycleRoot, isFocused, isLocationByPlatform, isOpaque, isShowing, isValidateRoot, pack, paint, postEvent, processEvent, processWindowFocusEvent, processWindowStateEvent, removeWindowFocusListener, removeWindowListener, removeWindowStateListener, reshape, setAlwaysOnTop, setAutoRequestFocus, setBounds, setBounds, setCursor, setFocusableWindowState, setFocusCycleRoot, setIconImages, setLocation, setLocation, setLocationByPlatform, setLocationRelativeTo, setMinimumSize, setModalExclusionType, setSize, setSize, setType, setVisible, show, toBack, toFront`
    - ### Methods inherited from class java.awt.Container

      `add, add, add, add, add, addContainerListener, applyComponentOrientation, areFocusTraversalKeysSet, countComponents, deliverEvent, doLayout, findComponentAt, findComponentAt, getAlignmentX, getAlignmentY, getComponent, getComponentAt, getComponentAt, getComponentCount, getComponents, getComponentZOrder, getContainerListeners, getFocusTraversalPolicy, getInsets, getLayout, getMaximumSize, getMinimumSize, getMousePosition, getPreferredSize, insets, invalidate, isAncestorOf, isFocusCycleRoot, isFocusTraversalPolicyProvider, isFocusTraversalPolicySet, layout, list, list, locate, minimumSize, paintComponents, preferredSize, print, printComponents, processContainerEvent, remove, removeAll, removeContainerListener, setComponentZOrder, setFocusTraversalKeys, setFocusTraversalPolicy, setFocusTraversalPolicyProvider, setFont, transferFocusDownCycle, validate, validateTree`
    - ### Methods inherited from class java.awt.Component

      `action, add, addComponentListener, addFocusListener, addHierarchyBoundsListener, addHierarchyListener, addInputMethodListener, addKeyListener, addMouseListener, addMouseMotionListener, addMouseWheelListener, bounds, checkImage, checkImage, coalesceEvents, contains, contains, createImage, createImage, createVolatileImage, createVolatileImage, disable, disableEvents, dispatchEvent, enable, enable, enableEvents, enableInputMethods, firePropertyChange, firePropertyChange, firePropertyChange, firePropertyChange, firePropertyChange, firePropertyChange, firePropertyChange, firePropertyChange, firePropertyChange, getBaseline, getBaselineResizeBehavior, getBounds, getBounds, getColorModel, getComponentListeners, getComponentOrientation, getCursor, getDropTarget, getFocusListeners, getFocusTraversalKeysEnabled, getFont, getFontMetrics, getForeground, getGraphicsConfiguration, getHeight, getHierarchyBoundsListeners, getHierarchyListeners, getIgnoreRepaint, getInputMethodListeners, getInputMethodRequests, getKeyListeners, getLocation, getLocation, getLocationOnScreen, getMouseListeners, getMouseMotionListeners, getMousePosition, getMouseWheelListeners, getName, getParent, getPeer, getPropertyChangeListeners, getPropertyChangeListeners, getSize, getSize, getTreeLock, getWidth, getX, getY, gotFocus, handleEvent, hasFocus, imageUpdate, inside, isBackgroundSet, isCursorSet, isDisplayable, isDoubleBuffered, isEnabled, isFocusable, isFocusOwner, isFocusTraversable, isFontSet, isForegroundSet, isLightweight, isMaximumSizeSet, isMinimumSizeSet, isPreferredSizeSet, isValid, isVisible, keyDown, keyUp, list, list, list, location, lostFocus, mouseDown, mouseDrag, mouseEnter, mouseExit, mouseMove, mouseUp, move, nextFocus, paintAll, prepareImage, prepareImage, printAll, processComponentEvent, processFocusEvent, processHierarchyBoundsEvent, processHierarchyEvent, processInputMethodEvent, processKeyEvent, processMouseEvent, processMouseMotionEvent, processMouseWheelEvent, removeComponentListener, removeFocusListener, removeHierarchyBoundsListener, removeHierarchyListener, removeInputMethodListener, removeKeyListener, removeMouseListener, removeMouseMotionListener, removeMouseWheelListener, removePropertyChangeListener, removePropertyChangeListener, repaint, repaint, repaint, requestFocus, requestFocus, requestFocusInWindow, requestFocusInWindow, resize, resize, revalidate, setComponentOrientation, setDropTarget, setEnabled, setFocusable, setFocusTraversalKeysEnabled, setForeground, setIgnoreRepaint, setLocale, setMaximumSize, setName, setPreferredSize, show, size, toString, transferFocus, transferFocusBackward, transferFocusUpCycle`
    - ### Methods inherited from class java.lang.Object

      `clone, equals, finalize, getClass, hashCode, notify, notifyAll, wait, wait, wait`
    - ### Methods inherited from interface java.awt.MenuContainer

      `getFont, postEvent`

- - ### Constructor Detail


    - #### NNFrame

      ```
      public NNFrame(GraphPanel p)
      ```
  - ### Method Detail


    - #### actionPerformed

      ```
      public void actionPerformed(java.awt.event.ActionEvent e)
      ```

      **Specified by:**
      :   `actionPerformed` in interface `java.awt.event.ActionListener`


    - #### mouseClicked

      ```
      public void mouseClicked(java.awt.event.MouseEvent arg0)
      ```

      **Specified by:**
      :   `mouseClicked` in interface `java.awt.event.MouseListener`


    - #### mouseEntered

      ```
      public void mouseEntered(java.awt.event.MouseEvent arg0)
      ```

      **Specified by:**
      :   `mouseEntered` in interface `java.awt.event.MouseListener`


    - #### mouseExited

      ```
      public void mouseExited(java.awt.event.MouseEvent arg0)
      ```

      **Specified by:**
      :   `mouseExited` in interface `java.awt.event.MouseListener`


    - #### mousePressed

      ```
      public void mousePressed(java.awt.event.MouseEvent arg0)
      ```

      **Specified by:**
      :   `mousePressed` in interface `java.awt.event.MouseListener`


    - #### mouseReleased

      ```
      public void mouseReleased(java.awt.event.MouseEvent arg0)
      ```

      **Specified by:**
      :   `mouseReleased` in interface `java.awt.event.MouseListener`


    - #### menuCanceled

      ```
      public void menuCanceled(javax.swing.event.MenuEvent e)
      ```

      **Specified by:**
      :   `menuCanceled` in interface `javax.swing.event.MenuListener`


    - #### menuDeselected

      ```
      public void menuDeselected(javax.swing.event.MenuEvent e)
      ```

      **Specified by:**
      :   `menuDeselected` in interface `javax.swing.event.MenuListener`


    - #### menuSelected

      ```
      public void menuSelected(javax.swing.event.MenuEvent e)
      ```

      **Specified by:**
      :   `menuSelected` in interface `javax.swing.event.MenuListener`


- Package
- Class
- Tree
- Deprecated
- Index
- Help

- Prev Class
- Next Class

- Frames
- No Frames

- All Classes

- Summary:
- Nested |
- Field |
- Constr |
- Method

- Detail:
- Field |
- Constr |
- Method
