# Supplementary material for: Automatic modular design of robot swarms using behavior trees as a control architecture
Source: PeerJ Comput Sci. 2020 Nov 9;6:e314. doi: 10.7717/peerj-cs.314 (PMC7924474; doi:10.7717/peerj-cs.314)
Supplement: Supplemental Information 3 [file peerj-cs-06-314-s003.zip › NEAT-private-master/misc/config/NetworkGraph/doc.html/Node.html]

Node


JavaScript is disabled on your browser.


- Package
- Class
- Tree
- Deprecated
- Index
- Help

- Prev Class
- Next Class

- Frames
- No Frames

- All Classes

- Summary:
- Nested |
- Field |
- Constr |
- Method

- Detail:
- Field |
- Constr |
- Method


## Class Node

- java.lang.Object
- - Node

- All Implemented Interfaces:
  :   INode

  ---

    

  ```
  public class Node
  extends java.lang.Object
  implements INode
  ```

  Node Class.

- - ### Nested Class Summary

    - ### Nested classes/interfaces inherited from interface INode

      `INode.Type`
  - ### Constructor Summary

    Constructors

    | Constructor and Description |
    | `Node(int x, int y, java.lang.String name)` |
    | `Node(java.lang.String name)` |
  - ### Method Summary

    Methods

    | Modifier and Type | Method and Description |
    | `java.awt.Color` | `getColor()` Gets the color of the node. |
    | `java.lang.String` | `getName()` Gets the name of the node. |
    | `int` | `getR()` Gets the radius of the node. |
    | `static int` | `getRadius()` |
    | `int` | `getScale()` Gets the scale of the node. |
    | `INode.Type` | `getType()` Gets the type of the node: input, hidden, output. |
    | `int` | `getX()` Gets the position x of the node. |
    | `int` | `getY()` Gets the position y of the node. |
    | `void` | `setColor(java.awt.Color color)` Sets the color of the node. |
    | `void` | `setName(java.lang.String name)` Sets the name of the node. |
    | `void` | `setPosition(int x, int y)` Sets the position (x,y) of the node. |
    | `void` | `setScale(int scale)` Sets the scale of all nodes. |
    | `void` | `setType(INode.Type type)` Sets the type of the node: input, hidden, output. |

    - ### Methods inherited from class java.lang.Object

      `clone, equals, finalize, getClass, hashCode, notify, notifyAll, toString, wait, wait, wait`

- - ### Constructor Detail


    - #### Node

      ```
      public Node(java.lang.String name)
      ```


    - #### Node

      ```
      public Node(int x,
          int y,
          java.lang.String name)
      ```
  - ### Method Detail


    - #### setPosition

      ```
      public void setPosition(int x,
                     int y)
      ```

      **Description copied from interface: `INode`**

      Sets the position (x,y) of the node.

      **Specified by:**
      :   `setPosition` in interface `INode`

      Parameters:
      :   `x` - position x.
      :   `y` - position y.


    - #### setName

      ```
      public void setName(java.lang.String name)
      ```

      **Description copied from interface: `INode`**

      Sets the name of the node.

      **Specified by:**
      :   `setName` in interface `INode`

      Parameters:
      :   `name` - name of the node.


    - #### setColor

      ```
      public void setColor(java.awt.Color color)
      ```

      **Description copied from interface: `INode`**

      Sets the color of the node.

      **Specified by:**
      :   `setColor` in interface `INode`

      Parameters:
      :   `color` - color of the node.


    - #### setScale

      ```
      public void setScale(int scale)
      ```

      **Description copied from interface: `INode`**

      Sets the scale of all nodes.

      **Specified by:**
      :   `setScale` in interface `INode`

      Parameters:
      :   `scale` - scale of all nodes.


    - #### setType

      ```
      public void setType(INode.Type type)
      ```

      **Description copied from interface: `INode`**

      Sets the type of the node: input, hidden, output.

      **Specified by:**
      :   `setType` in interface `INode`


    - #### getX

      ```
      public int getX()
      ```

      **Description copied from interface: `INode`**

      Gets the position x of the node.

      **Specified by:**
      :   `getX` in interface `INode`

      Returns:
      :   position x


    - #### getY

      ```
      public int getY()
      ```

      **Description copied from interface: `INode`**

      Gets the position y of the node.

      **Specified by:**
      :   `getY` in interface `INode`

      Returns:
      :   position y


    - #### getR

      ```
      public int getR()
      ```

      **Description copied from interface: `INode`**

      Gets the radius of the node.

      **Specified by:**
      :   `getR` in interface `INode`

      Returns:
      :   position y


    - #### getRadius

      ```
      public static int getRadius()
      ```


    - #### getName

      ```
      public java.lang.String getName()
      ```

      **Description copied from interface: `INode`**

      Gets the name of the node.

      **Specified by:**
      :   `getName` in interface `INode`

      Returns:
      :   name


    - #### getColor

      ```
      public java.awt.Color getColor()
      ```

      **Description copied from interface: `INode`**

      Gets the color of the node.

      **Specified by:**
      :   `getColor` in interface `INode`

      Returns:
      :   color


    - #### getScale

      ```
      public int getScale()
      ```

      **Description copied from interface: `INode`**

      Gets the scale of the node.

      **Specified by:**
      :   `getScale` in interface `INode`

      Returns:
      :   color


    - #### getType

      ```
      public INode.Type getType()
      ```

      **Description copied from interface: `INode`**

      Gets the type of the node: input, hidden, output.

      **Specified by:**
      :   `getType` in interface `INode`

      Returns:
      :   type


- Package
- Class
- Tree
- Deprecated
- Index
- Help

- Prev Class
- Next Class

- Frames
- No Frames

- All Classes

- Summary:
- Nested |
- Field |
- Constr |
- Method

- Detail:
- Field |
- Constr |
- Method
