# Supplementary material for: Automatic modular design of robot swarms using behavior trees as a control architecture
Source: PeerJ Comput Sci. 2020 Nov 9;6:e314. doi: 10.7717/peerj-cs.314 (PMC7924474; doi:10.7717/peerj-cs.314)
Supplement: Supplemental Information 3 [file peerj-cs-06-314-s003.zip › NEAT-private-master/misc/config/NetworkGraph/doc.html/allclasses-frame.html]

All Classes


# All Classes

- Edge
- Graph
- GraphPanel
- *IEdge*
- *IGraph*
- *INode*
- INode.Type
- Main
- NNFrame
- Node
