# Supplementary material for: Automatic modular design of robot swarms using behavior trees as a control architecture
Source: PeerJ Comput Sci. 2020 Nov 9;6:e314. doi: 10.7717/peerj-cs.314 (PMC7924474; doi:10.7717/peerj-cs.314)
Supplement: Supplemental Information 3 [file peerj-cs-06-314-s003.zip › NEAT-private-master/misc/config/NetworkGraph/doc.html/index-all.html]

Index


JavaScript is disabled on your browser.


- Package
- Class
- Tree
- Deprecated
- Index
- Help

- Prev
- Next

- Frames
- No Frames

- All Classes

A C E G I M N P R S V Z 


## A

actionPerformed(ActionEvent) - Method in class GraphPanel


actionPerformed(ActionEvent) - Method in class NNFrame


addEdge(IEdge) - Method in class Graph


addEdge(ArrayList<IEdge>) - Method in class Graph


addEdge(IEdge) - Method in class GraphPanel


addEdge(IEdge) - Method in interface IGraph
:   Adds a new edge (connecting 2 nodes) to the graph.

addEdge(ArrayList<IEdge>) - Method in interface IGraph
:   Adds a new list of edges to the graph.

addNode(INode) - Method in class Graph


addNode(ArrayList<INode>) - Method in class Graph


addNode() - Method in class GraphPanel


addNode(INode) - Method in interface IGraph
:   Adds a new node to the graph.

addNode(ArrayList<INode>) - Method in interface IGraph
:   Adds a new list of nodes to the graph.


## C

closeSocialGraph() - Method in class GraphPanel


contains(INode) - Method in class Edge


contains(INode) - Method in class Graph


contains(IEdge) - Method in class Graph


contains(INode) - Method in interface IEdge
:   Checks if the node is at the end of one side of the edge.

contains(INode) - Method in interface IGraph
:   Checks if the graph contains a specific node.

contains(IEdge) - Method in interface IGraph
:   Checks if the graph contains a specific edge connecting 2 nodes.


## E

Edge - Class in <Unnamed>
:   Edge Class.

Edge(INode, INode) - Constructor for class Edge


Edge(INode, INode, int) - Constructor for class Edge


export(String, String) - Method in class GraphPanel


## G

getColor() - Method in interface INode
:   Gets the color of the node.

getColor() - Method in class Node


getEdge(int) - Method in class Graph


getEdge(int) - Method in interface IGraph
:   Gets the ith edge.

getListEdges() - Method in class Graph


getListEdges() - Method in interface IGraph
:   Gets the list of all edges.

getListEdgesSize() - Method in class Graph


getListEdgesSize() - Method in interface IGraph
:   Gets the size of the list of all edges.

getListNodes() - Method in class Graph


getListNodes() - Method in interface IGraph
:   Gets the list of all nodes.

getListNodesSize() - Method in class Graph


getListNodesSize() - Method in interface IGraph
:   Gets the size of the list of all nodes.

getName() - Method in interface INode
:   Gets the name of the node.

getName() - Method in class Node


getNode(int) - Method in class Graph


getNode(int) - Method in interface IGraph
:   Gets the ith node.

getNode1() - Method in class Edge


getNode1() - Method in interface IEdge
:   Gets the node which is at the end of one side of the edge.

getNode2() - Method in class Edge


getNode2() - Method in interface IEdge
:   Gets the node which is at the end of the other side of the edge.

getR() - Method in interface INode
:   Gets the radius of the node.

getR() - Method in class Node


getRadius() - Static method in class Node


getScale() - Method in interface INode
:   Gets the scale of the node.

getScale() - Method in class Node


getType() - Method in interface INode
:   Gets the type of the node: input, hidden, output.

getType() - Method in class Node


getWeight() - Method in class Edge


getWeight() - Method in interface IEdge
:   Gets the weight/cost of the edge.

getX() - Method in interface INode
:   Gets the position x of the node.

getX() - Method in class Node


getY() - Method in interface INode
:   Gets the position y of the node.

getY() - Method in class Node


Graph - Class in <Unnamed>
:   Graph Class.

Graph(ArrayList<INode>, ArrayList<IEdge>) - Constructor for class Graph


Graph() - Constructor for class Graph


GraphPanel - Class in <Unnamed>
:   GraphPanel Class.

GraphPanel(IGraph) - Constructor for class GraphPanel


## I

IEdge - Interface in <Unnamed>
:   Edge Interface.

IGraph - Interface in <Unnamed>
:   Graph Interface.

INode - Interface in <Unnamed>
:   Node Interface.

INode.Type - Enum in <Unnamed>


## M

Main - Class in <Unnamed>
:   Main Class.

Main() - Constructor for class Main


main(String[]) - Static method in class Main


menuCanceled(MenuEvent) - Method in class NNFrame


menuDeselected(MenuEvent) - Method in class NNFrame


menuSelected(MenuEvent) - Method in class NNFrame


mouseClicked(MouseEvent) - Method in class GraphPanel


mouseClicked(MouseEvent) - Method in class NNFrame


mouseDragged(MouseEvent) - Method in class GraphPanel


mouseEntered(MouseEvent) - Method in class GraphPanel


mouseEntered(MouseEvent) - Method in class NNFrame


mouseExited(MouseEvent) - Method in class GraphPanel


mouseExited(MouseEvent) - Method in class NNFrame


mouseMoved(MouseEvent) - Method in class GraphPanel


mousePressed(MouseEvent) - Method in class GraphPanel


mousePressed(MouseEvent) - Method in class NNFrame


mouseReleased(MouseEvent) - Method in class GraphPanel


mouseReleased(MouseEvent) - Method in class NNFrame


## N

NNFrame - Class in <Unnamed>
:   NNFrame (=Neural Network Frame) Class.

NNFrame(GraphPanel) - Constructor for class NNFrame


Node - Class in <Unnamed>
:   Node Class.

Node(String) - Constructor for class Node


Node(int, int, String) - Constructor for class Node


## P

paintComponent(Graphics) - Method in class GraphPanel


## R

removeEdge(IEdge) - Method in class Graph


removeEdge(int) - Method in class Graph


removeEdge(INode) - Method in class Graph


removeEdge(IEdge) - Method in class GraphPanel


removeEdge(IEdge) - Method in interface IGraph
:   Removes an edge from the graph.

removeEdge(int) - Method in interface IGraph
:   Removes the ith edge from the graph.

removeEdge(INode) - Method in interface IGraph
:   Removes all edges which have a common node at one extremity.

removeNode(INode) - Method in class Graph


removeNode(int) - Method in class Graph


removeNode() - Method in class GraphPanel


removeNode(INode) - Method in interface IGraph
:   Removes a node from the graph.

removeNode(int) - Method in interface IGraph
:   Removes the ith node from the graph.


## S

setColor(Color) - Method in interface INode
:   Sets the color of the node.

setColor(Color) - Method in class Node


setListNodeAndListEdge(ArrayList<INode>, ArrayList<IEdge>) - Method in class Graph


setName(String) - Method in interface INode
:   Sets the name of the node.

setName(String) - Method in class Node


setPosition(int, int) - Method in interface INode
:   Sets the position (x,y) of the node.

setPosition(int, int) - Method in class Node


setPositionToAllNodes(int, int) - Method in class Graph


setPositionToAllNodes(int, int) - Method in interface IGraph
:   Sets the position of all nodes in the graph.

setScale(int) - Method in interface INode
:   Sets the scale of all nodes.

setScale(int) - Method in class Node


setType(INode.Type) - Method in interface INode
:   Sets the type of the node: input, hidden, output.

setType(INode.Type) - Method in class Node


setWeight(int) - Method in class Edge


setWeight(int) - Method in interface IEdge
:   Sets the weight/cost of the edge.


## V

valueOf(String) - Static method in enum INode.Type
:   Returns the enum constant of this type with the specified name.

values() - Static method in enum INode.Type
:   Returns an array containing the constants of this enum type, in
    the order they are declared.


## Z

zoomIn() - Method in class GraphPanel


zoomOut() - Method in class GraphPanel

A C E G I M N P R S V Z

- Package
- Class
- Tree
- Deprecated
- Index
- Help

- Prev
- Next

- Frames
- No Frames

- All Classes
