# Supplementary material for: Automatic modular design of robot swarms using behavior trees as a control architecture
Source: PeerJ Comput Sci. 2020 Nov 9;6:e314. doi: 10.7717/peerj-cs.314 (PMC7924474; doi:10.7717/peerj-cs.314)
Supplement: Supplemental Information 3 [file peerj-cs-06-314-s003.zip › NEAT-private-master/misc/config/NetworkGraph/doc.html/index.html]

Generated Documentation (Untitled)


<noscript>
<div>JavaScript is disabled on your browser.</div>
</noscript>
<h2>Frame Alert</h2>
<p>This document is designed to be viewed using the frames feature. If you see this message, you are using a non-frame-capable web client. Link to <a href="Edge.html">Non-frame version</a>.</p>
