# Supplementary material for: Automatic modular design of robot swarms using behavior trees as a control architecture
Source: PeerJ Comput Sci. 2020 Nov 9;6:e314. doi: 10.7717/peerj-cs.314 (PMC7924474; doi:10.7717/peerj-cs.314)
Supplement: Supplemental Information 3 [file peerj-cs-06-314-s003.zip › NEAT-private-master/misc/config/NetworkGraph/doc.html/package-summary.html]

JavaScript is disabled on your browser.


- Package
- Class
- Tree
- Deprecated
- Index
- Help

- Prev Package
- Next Package

- Frames
- No Frames

- All Classes

# Package <Unnamed>

- Interface Summary

  | Interface | Description |
  |  |  |
  | --- | --- |
  | IEdge | Edge Interface. |
  | IGraph | Graph Interface. |
  | INode | Node Interface. |
- Class Summary

  | Class | Description |
  |  |  |
  | --- | --- |
  | Edge | Edge Class. |
  | Graph | Graph Class. |
  | GraphPanel | GraphPanel Class. |
  | Main | Main Class. |
  | NNFrame | NNFrame (=Neural Network Frame) Class. |
  | Node | Node Class. |
- Enum Summary

  | Enum | Description |
  |  |  |
  | --- | --- |
  | INode.Type |  |

- Package
- Class
- Tree
- Deprecated
- Index
- Help

- Prev Package
- Next Package

- Frames
- No Frames

- All Classes
