# Supplementary material for: Automatic modular design of robot swarms using behavior trees as a control architecture
Source: PeerJ Comput Sci. 2020 Nov 9;6:e314. doi: 10.7717/peerj-cs.314 (PMC7924474; doi:10.7717/peerj-cs.314)
Supplement: Supplemental Information 3 [file peerj-cs-06-314-s003.zip › NEAT-private-master/misc/config/NetworkGraph/doc.html/package-tree.html]

 Class Hierarchy


JavaScript is disabled on your browser.


- Package
- Class
- Tree
- Deprecated
- Index
- Help

- Prev
- Next

- Frames
- No Frames

- All Classes

# Hierarchy For Package <Unnamed>

## Class Hierarchy

- java.lang.Object
  - java.awt.Component (implements java.awt.image.ImageObserver, java.awt.MenuContainer, java.io.Serializable)
    - java.awt.Container
      - javax.swing.JComponent (implements java.io.Serializable)
        - javax.swing.JPanel (implements javax.accessibility.Accessible)
          - GraphPanel (implements java.awt.event.ActionListener, java.awt.event.MouseListener, java.awt.event.MouseMotionListener)
      - java.awt.Window (implements javax.accessibility.Accessible)
        - java.awt.Frame (implements java.awt.MenuContainer)
          - javax.swing.JFrame (implements javax.accessibility.Accessible, javax.swing.RootPaneContainer, javax.swing.WindowConstants)
            - NNFrame (implements java.awt.event.ActionListener, javax.swing.event.MenuListener, java.awt.event.MouseListener)
  - Edge (implements IEdge)
  - Graph (implements IGraph)
  - Main
  - Node (implements INode)

## Interface Hierarchy

- IEdge
- IGraph
- INode

## Enum Hierarchy

- java.lang.Object
  - java.lang.Enum<E> (implements java.lang.Comparable<T>, java.io.Serializable)
    - INode.Type

- Package
- Class
- Tree
- Deprecated
- Index
- Help

- Prev
- Next

- Frames
- No Frames

- All Classes
