# Supplementary material for: Automatic modular design of robot swarms using behavior trees as a control architecture
Source: PeerJ Comput Sci. 2020 Nov 9;6:e314. doi: 10.7717/peerj-cs.314 (PMC7924474; doi:10.7717/peerj-cs.314)
Supplement: Supplemental Information 3 [file peerj-cs-06-314-s003.zip › NEAT-private-master/misc/config/NetworkGraph/doc.html/serialized-form.html]

Serialized Form


JavaScript is disabled on your browser.


- Package
- Class
- Tree
- Deprecated
- Index
- Help

- Prev
- Next

- Frames
- No Frames

- All Classes

# Serialized Form

- ## Package &lt;Unnamed&gt;

  - ### Class GraphPanel extends javax.swing.JPanel implements Serializable

    - ### Serialized Fields

      - #### graph

        ```
        IGraph graph
        ```
      - #### listNodeSize

        ```
        int listNodeSize
        ```
      - #### listEdgeSize

        ```
        int listEdgeSize
        ```
      - #### nodeDragged

        ```
        INode nodeDragged
        ```
      - #### selectedNode

        ```
        INode[] selectedNode
        ```
      - #### repaint

        ```
        boolean repaint
        ```
      - #### repaintAll

        ```
        boolean repaintAll
        ```
      - #### mouseX

        ```
        int mouseX
        ```
      - #### mouseY

        ```
        int mouseY
        ```
      - #### colorTable

        ```
        java.lang.String[] colorTable
        ```
      - #### popUpMenu

        ```
        javax.swing.JPopupMenu popUpMenu
        ```
      - #### menuItemList

        ```
        java.util.ArrayList<E> menuItemList
        ```
      - #### number

        ```
        int number
        ```
      - #### firstTime

        ```
        boolean firstTime
        ```
  - ### Class NNFrame extends javax.swing.JFrame implements Serializable

    - ### Serialized Fields

      - #### panel

        ```
        GraphPanel panel
        ```

- Package
- Class
- Tree
- Deprecated
- Index
- Help

- Prev
- Next

- Frames
- No Frames

- All Classes
