# Supplementary material for: Automatic modular design of robot swarms using behavior trees as a control architecture
Source: PeerJ Comput Sci. 2020 Nov 9;6:e314. doi: 10.7717/peerj-cs.314 (PMC7924474; doi:10.7717/peerj-cs.314)

Problem Statement

We assume that we have 20 robots in the prescribed arena. There are two spots in circle shape on the floor. One is black and placed at $[x, y] = [0, -0.60]$ with radius 0.30. The second one is white and placed at $[x, y] = [0, 0.60]$ with radius 0.30. There is also a light source at $[x, y] = [0, 1.25]$. Note that all distances are in meter. Figure below shows the scheme of the arena with the spots, robots, and light source.

The objective is to design a controller that maximizes the number of robots on the white spot. In particular, we define the objective function as the summation of the number of robots placed on the white spot at each time step over the experiment time, that is: $J = \sum_{k=1}^{t_f} N_w(k)$ where k is the time step, t_f is the final time step, and $N_w(k)$ is the number of robots on the white spot at time step k.

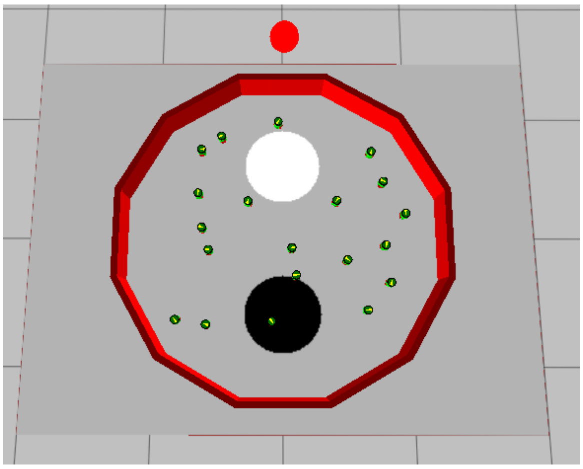

Supplement: Supplemental Information 3 [file peerj-cs-06-314-s003.zip › NEAT-private-master/misc/old_loop_functions/aac/Problem_statement.pdf]
