# Supplementary material for: Automatic modular design of robot swarms using behavior trees as a control architecture
Source: PeerJ Comput Sci. 2020 Nov 9;6:e314. doi: 10.7717/peerj-cs.314 (PMC7924474; doi:10.7717/peerj-cs.314)
Supplement: Supplemental Information 3 [file peerj-cs-06-314-s003.zip › NEAT-private-master/misc/old_loop_functions/cfa/Problem_statement.pdf]

Coverage with forbidden areas

The goal of this swarm behavior is to cover the entire arena except some *forbidden areas*. A *forbidden area* is an area in the arena where the robots cannot go.

The swarm must maximize the area that it covers with the exception of the forbidden areas. The distance between the robots must therfore be maximum outside the forbidden areas.

The objective function used to measure the controller efficiency is $f(d) = E[d]$ where $E[d]$ is the expected distance between a set of random points chosen inside the arena and their closest robots that are not in a forbidden area. The controller must minimize this function, i.e., $min_d E[d]$.

In the arena used for our experiments, we placed three *forbidden areas*. Each forbidden area is abstracted by a black circle of radius $r = 30\ cm$. The circles are centered in $c1 = (0.5, 0.5), c_2 = (0.5, -0.5)$ and $c_3 = (-0.5, 0)$.
