# Supplementary material for: Automatic modular design of robot swarms using behavior trees as a control architecture
Source: PeerJ Comput Sci. 2020 Nov 9;6:e314. doi: 10.7717/peerj-cs.314 (PMC7924474; doi:10.7717/peerj-cs.314)
Supplement: Supplemental Information 3 [file peerj-cs-06-314-s003.zip › NEAT-private-master/misc/old_loop_functions/spc/Problem_statement.pdf]

SURFACE AND PERIMETER COVERAGE

1. Description

In the arena 2 colored patches have been added. The first patch is a white square with the center in (-0.6,0) and the sides 0.6 m long. The second patch is a black circle with the center in (0.6,0) and a radius of 0.3 m. At the beginning of the experiment the 20 robots are placed randomly over the entire area of the arena. The goal of the robots is to cover as much area of the white square as possible and as much perimeter of the black circle as possible.

2. Evaluation

The evaluation is performed at the end of the experiment. The evaluation function samples 1000 random points within the white area, measures the distance of each point from the closest robot inside the white area and finally computes the average of these 1000 distances. The same process is repeated for the black patch, this time sampling 1000 points on its perimeter and considering only robots on the perimeter (a robot is considered on the perimeter when the circle surrounding its round body intersects the perimeter of the black patch). Once the average distance for the coverage of the white area (d_A) and the average distance for the coverage of the black perimeter (d_P) have been computed, the objective function to *minimize* is the following:

$$\hat{d} = \frac{d_A}{{d_A}^*} + \frac{d_P}{{d_P}^*} \tag{1}$$

Where ${d_A}^*$ and ${d_P}^*$ are the optimal values of the distances for the coverage of area and perimeter, respectively. These two values were computed by distributing 9 robots equally spaced over the white area and 9 robots equally spaced along the black perimeter and finally calculating the average distances as described above.
