# Supplementary figures and images for: Automatic modular design of robot swarms using behavior trees as a control architecture
Source: PeerJ Comput Sci. 2020 Nov 9;6:e314. doi: 10.7717/peerj-cs.314 (PMC7924474; doi:10.7717/peerj-cs.314)

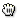

Supplement: Supplemental Information 3 [file peerj-cs-06-314-s003.zip › NEAT-private-master/misc/config/NetworkGraph/SocialGraph/socialGraph/images/closed.png]

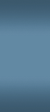

Supplement: Supplemental Information 3 [file peerj-cs-06-314-s003.zip › NEAT-private-master/misc/config/NetworkGraph/doc.html/resources/background.gif]

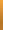

Supplement: Supplemental Information 3 [file peerj-cs-06-314-s003.zip › NEAT-private-master/misc/config/NetworkGraph/doc.html/resources/tab.gif]

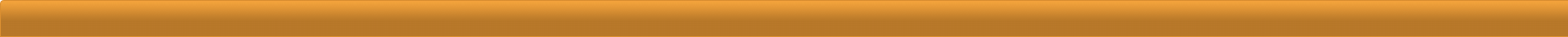

Supplement: Supplemental Information 3 [file peerj-cs-06-314-s003.zip › NEAT-private-master/misc/config/NetworkGraph/doc.html/resources/titlebar.gif]

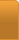

Supplement: Supplemental Information 3 [file peerj-cs-06-314-s003.zip › NEAT-private-master/misc/config/NetworkGraph/doc.html/resources/titlebar_end.gif]

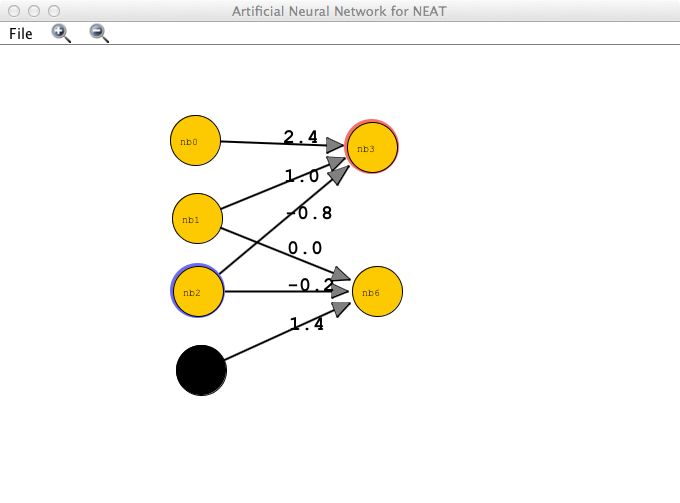

Supplement: Supplemental Information 3 [file peerj-cs-06-314-s003.zip › NEAT-private-master/misc/config/NetworkGraph/examples/drawingTool.png]

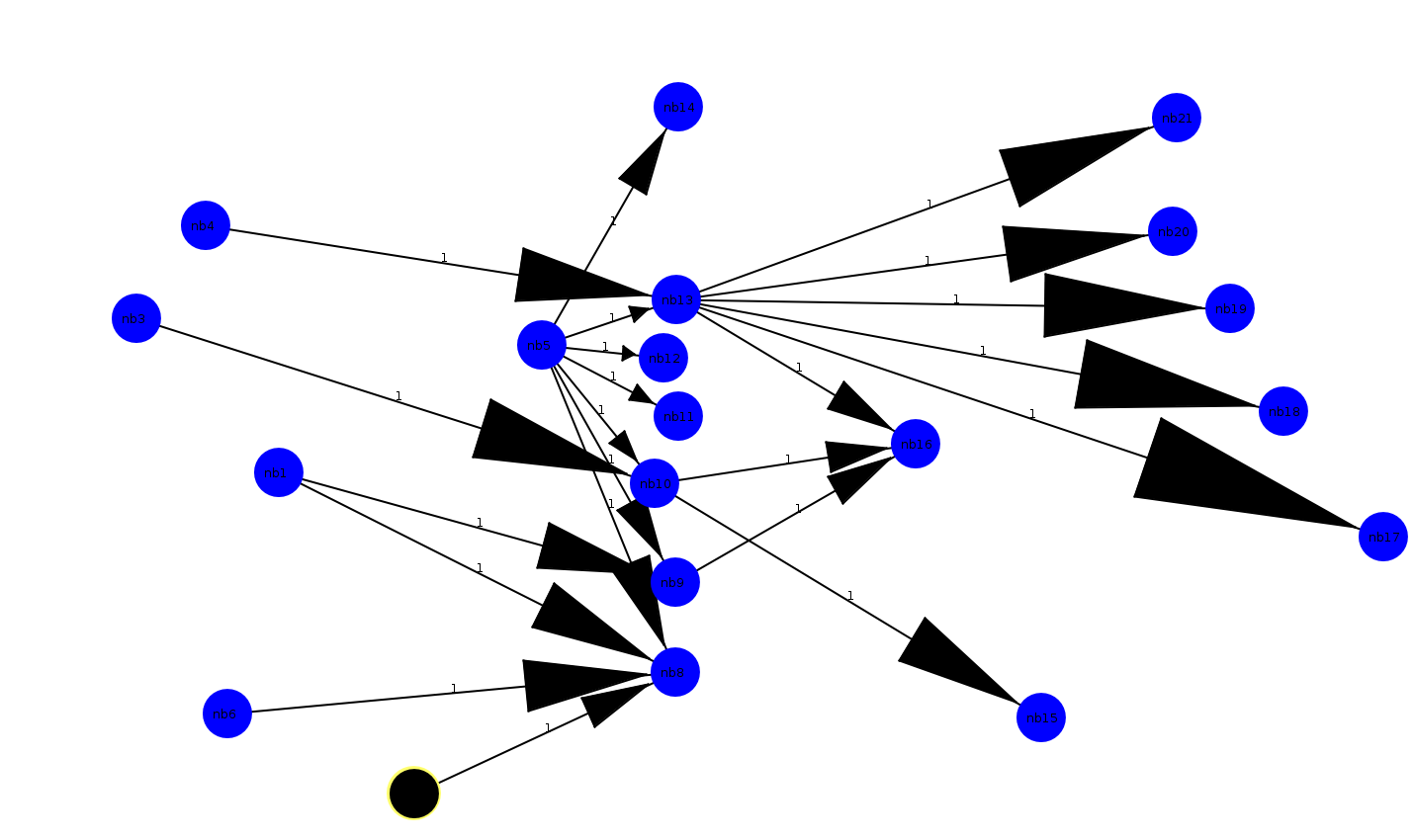

Supplement: Supplemental Information 3 [file peerj-cs-06-314-s003.zip › NEAT-private-master/misc/config/NetworkGraph/examples/v1.png]

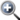

Supplement: Supplemental Information 3 [file peerj-cs-06-314-s003.zip › NEAT-private-master/misc/config/NetworkGraph/images/zoomin.png]

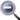

Supplement: Supplemental Information 3 [file peerj-cs-06-314-s003.zip › NEAT-private-master/misc/config/NetworkGraph/images/zoomout.png]

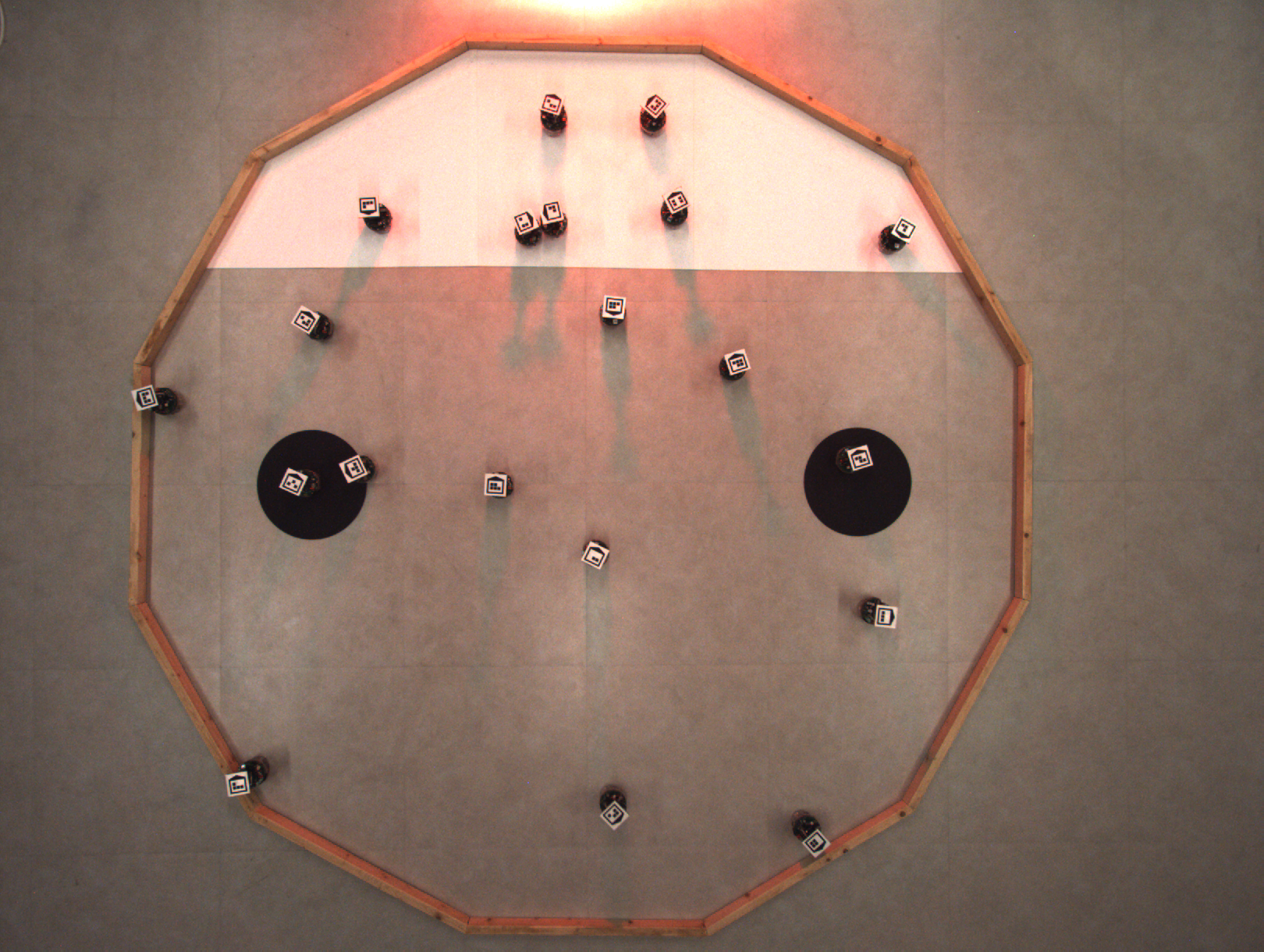

Supplement: Supplemental Information 4 [file peerj-cs-06-314-s004.zip › AutoMoDe-private-BehaviorTree/Experiments/Figures/Run_Foraging.bmp]

# \textsc{aggregation}

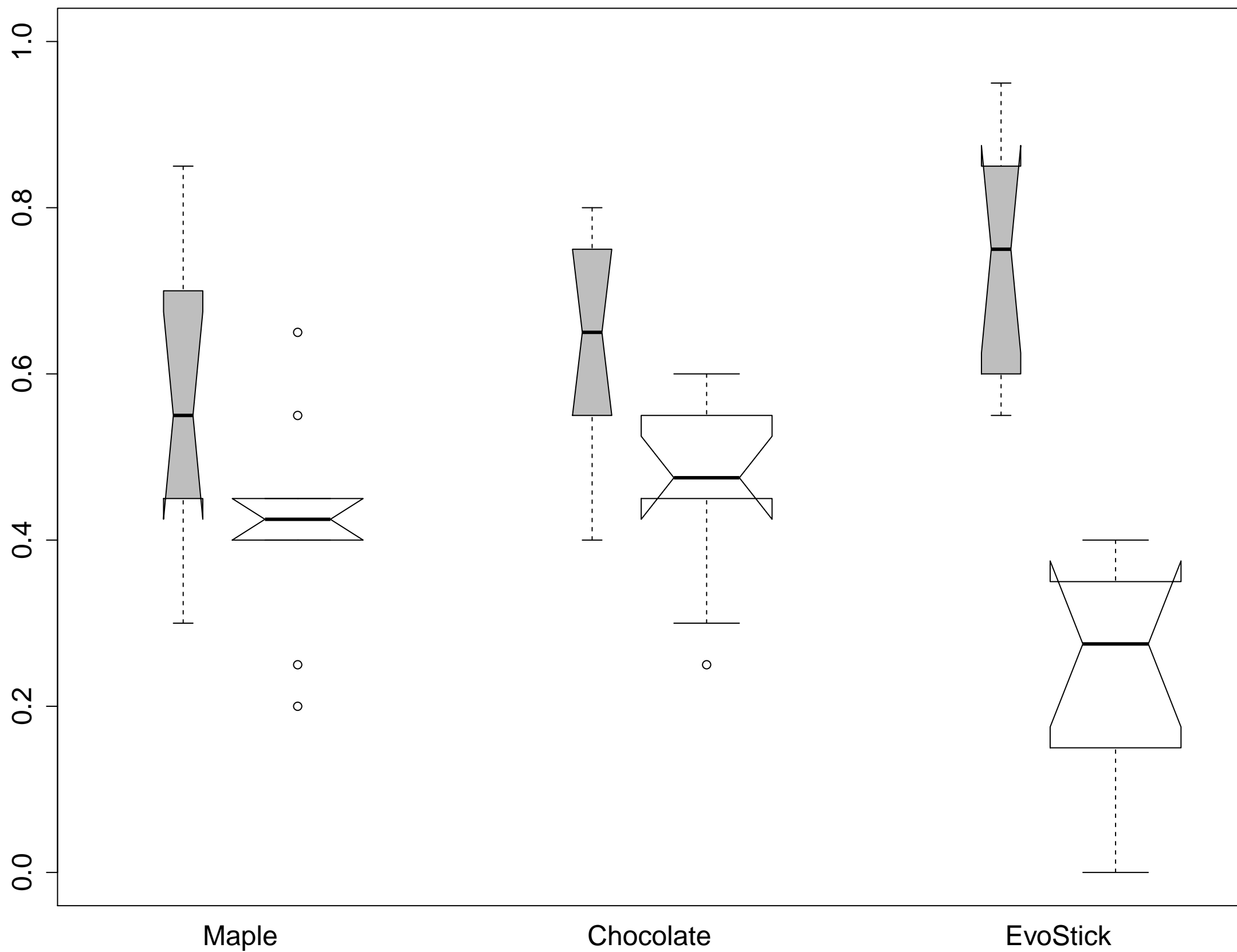

Supplement: Supplemental Information 4 [file peerj-cs-06-314-s004.zip › AutoMoDe-private-BehaviorTree/Experiments/Figures/agg2-one-eval.pdf]

# \textsc{foraging}

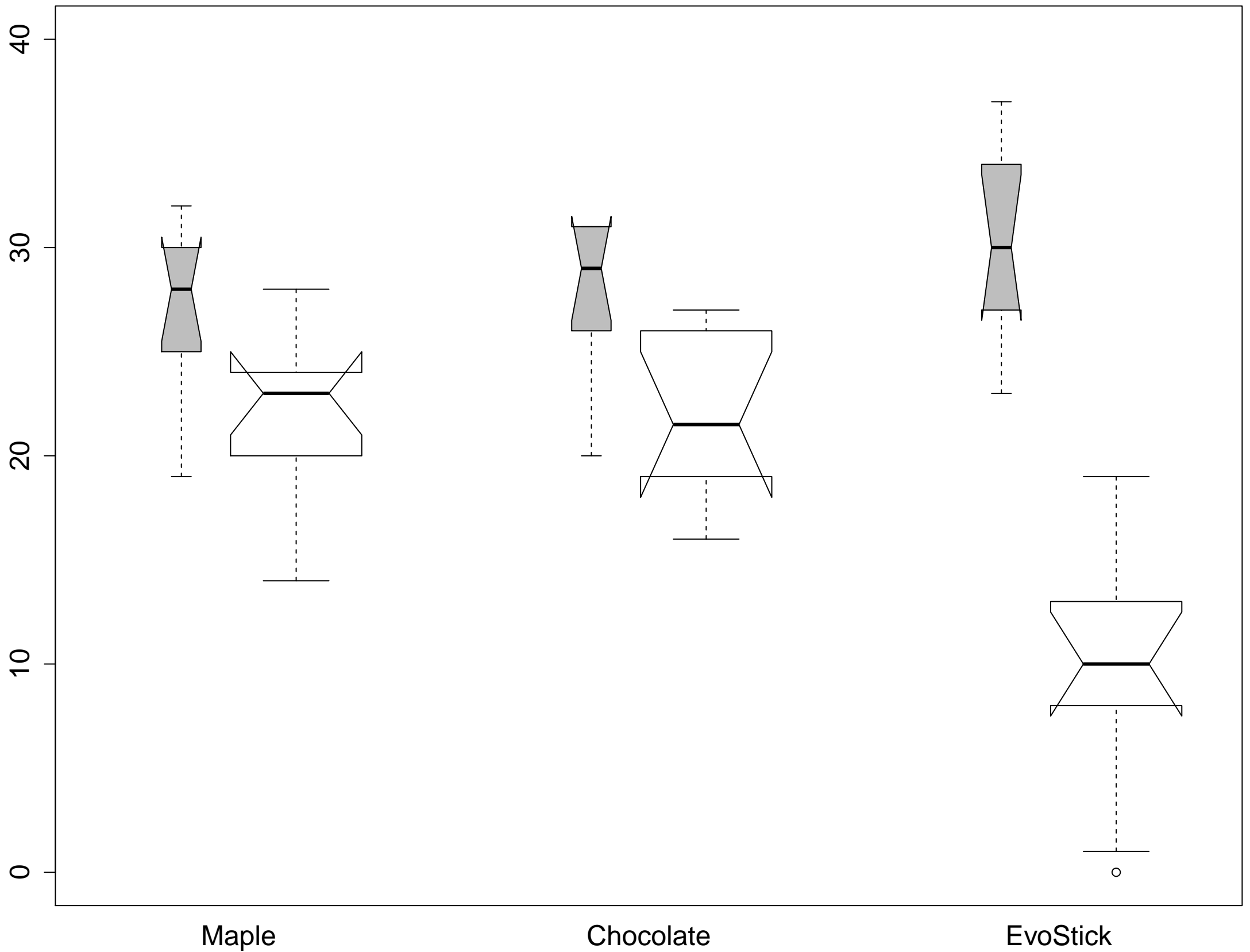

Supplement: Supplemental Information 4 [file peerj-cs-06-314-s004.zip › AutoMoDe-private-BehaviorTree/Experiments/Figures/forag-one-eval.pdf]
